# Clinical application value of hepatitis B virus basal core promoter 1762/1764 and GGTII and GGT in patients with HBV-DNA-positive primary liver cancer

**DOI:** 10.1097/MD.0000000000035699

**Published:** 2023-10-27

**Authors:** Shunhua Qiu, Lifen Jin, Dan Yang, Dewen Zhang

**Affiliations:** a Department of Clinical Laboratory, Zigong Third People’s Hospital, Sichuan, P.R.[aff_start] [/aff_end]China; b Department of pharmaceutical preparation, Zigong Third People’s Hospital, Sichuan, P.R.[aff_start] [/aff_end]China.

**Keywords:** gamma-glutamyl transpeptidase, GGTII, HBV base core promoter 1762/1764, primary liver cancer

## Abstract

**Background:**

Hepatitis B virus (HBV) is closely related to the occurrence and development of primary liver cancer (PLC). The early diagnosis of PLC is difficult. The study explored the clinical application value of the HBV gene basal core promoter (BCP) region 1762/1764 combined with gamma-glutamyl transpeptidase (GGT) and its isozyme II (GGTII) in PLC.

**Methods:**

From June 2017 to June 2021, 145 hepatitis B surface antigen-positive and HBV DNA-positive patients were enrolled in the Third People Hospital of Zigong. Of them, 67 were chronic hepatitis B (CHB) patients, 30 were liver cirrhosis patients, and 48 were patients with hepatitis B-associated PLC. The HBV BCP 1762/1764 mutation was detected through the amplification refractory mutation system fluorescence PCR method, and GGTII was detected using the double-antibody sandwich method.

**Results:**

The results showed that the serum GGT activity, GGTII level, aspartate aminotransferase (AST) activity, AST/alanine aminotransferase (ALT) ratio, GGT/ALT ratio, and GGT/AST ratio were significantly different between the PLC and CHB groups. Statistically significant differences in serum GGT activity, AST activity, and GGT/ALT ratio were observed between the PLC and LC groups. The BCP 1762/1764 mutation rate between the PLC and CHB groups was statistically significant. The GGTII level in the early PLC (stage I + II) group and the advanced PLC (stage III + IV) group was higher than that in the N-PLC group. Serum GGT activity in the early PLC and advanced PLC groups was higher than that in the N-PLC group. The area under the curve of the receiver operator characteristic curve of GGT and GGTII for diagnosing PLC was 0.775 (95% confidence interval [CI] [0.697, 0.854]) and 0.608 (95% CI [0.512, 0.704]), respectively. The area under curve of GGT and GGTII for diagnosing early PLC was 0.732 (95% CI [0.620, 0.845]) and 0.579 (95% CI [0.452, 0.706]), respectively.

**Conclusion:**

HBV gene BCP 1762/1764 mutation, GGT, and GGTII may be related to PLC occurrence. The HBV gene BCP region 1762/1764 combined with GGT has certain clinical diagnostic values for PLC and early PLC. However, GGTII is not a good indicator of early PLC and is more relevant to advanced PLC.

## 1. Introduction

Primary liver cancer (PLC) is among the most common malignancies worldwide, and its incidence and mortality rate have recently increased.^[1]^ Clinically, the early diagnosis of PLC is more difficult, and many confirmed PLC cases are already in the middle and late stages. The treatment effect and prognosis of PLC are poor. Therefore, indicators for early PLC diagnosis must be identified. The causes of PLC are very complex. PLC is often caused by the synergistic effect of multiple factors.

Hepatitis B virus (HBV) is closely related to PLC occurrence and development. China has the highest incidence of chronic hepatitis B (CHB) in the world, accounting for one-third of the total cases.^[2]^ In the process of chronic HBV continuous infection and HBV DNA replication in patients, the HBV gene is prone to mutation, so that the virus escapes immunity, causing greater harm to the body and may even lead to cancer.^[3–5]^ Mutations of HBV gene basal core promoter (BCP) region 1762/1764 are significantly associated with PLC occurrence.^[6]^ Alanine aminotransferase (ALT), gamma-glutamyl transpeptidase (GGT), and aspartate aminotransferase (AST) all increased in CHB patients, and GGT levels increased more significantly in PLC patients.^[7]^

PLC is mainly diagnosed using imaging, serum alpha-fetoprotein, pathology, and other methods. GGT isozyme II (GGTII) is also a crucial liver cancer marker. This parameter and low alpha-fetoprotein levels are important diagnostic markers of the early stage of PLC.^[8,9]^ The reason for the elevated GGTII levels in PLC patients has not been fully elaborated.

Therefore, this study investigated the relationship between gene mutation and GGT and GGT and GGTII in PLC of HBV DNA-positive patients. The relationship between HBV BCP1762/1764 and GGT and GGTII was analyzed from the perspective of gene variation and biochemical markers in the BCP region. The clinical value of combined detection of BCP1762/1764 mutation with GGT and GGTII in early PLC diagnosis was investigated.

## 2. Materials and methods

### 2.1. Study design and data collection

**Figure 1.**
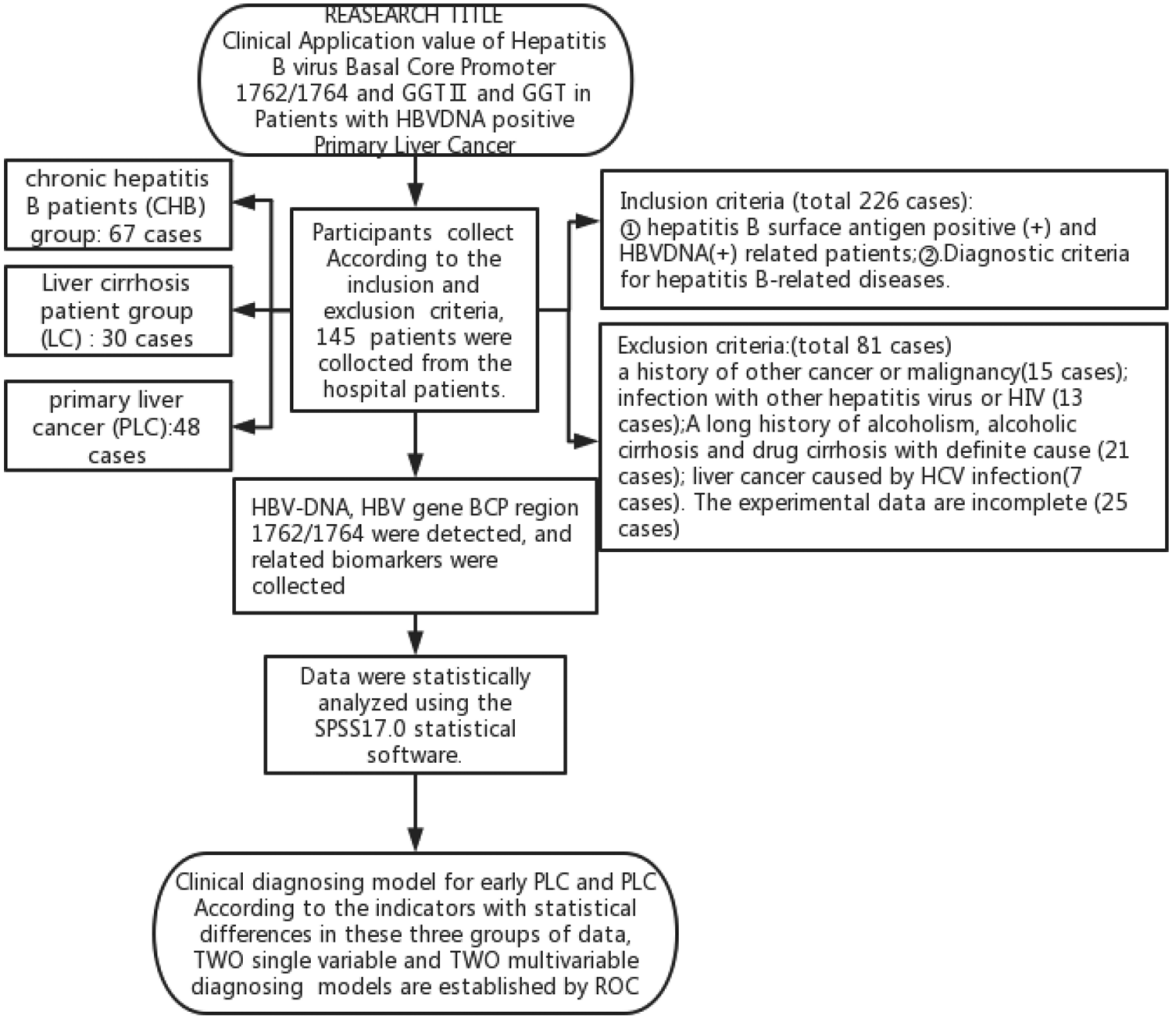
Schematic illustration of the study protocol.

Hepatitis B surface antigen-positive and HBV DNA-positive patients were randomly selected from among the outpatients and inpatients of the Third People Hospital of Zigong. The patients were selected from June 2017 to June 2021. After applying the exclusion criteria (exclusion of 56 cases) and discarding cases with incomplete data (25 cases), 145 cases were finally included in the study. These patients were divided into 3 groups: CHB group had 67 cases, including 41 male patients and 26 female patients with an average age of 55 ± 15.37 years; LC group had 30 cases, including 22 male patients and 8 female patients with an average age of 55.23 ± 8.23 years; and PLC group had 48 cases, including 42 male patients and 6 female patients with an average age of 59.79 ± 14.33 years. The patients with PLC were staged according to the TNM staging system of the American Joint Committee on Cancer: 10 patients (20.83%) were in stage I, 12 (25%) in stage II, 14 (29.17%) in stage III, and 12 (25%) in stage IV. The CHB and LC groups were collectively referred to as the non-PLC (N-PLC) group, patients with stage I and stage II PLC were assigned to the early PLC group, and patients with stage III and stage IV PLC were assigned to the advanced PLC group. Relevant biochemical indicators, namely ALT, AST, and GGT levels, were determined. The diagnostic criteria followed for hepatitis B-related diseases in the patients were based on the Guidelines for the Prevention and Treatment of CHB (2019 edition) and the Guidelines for the Diagnosis and Treatment of Primary Liver Cancer (2019 edition). Patients with a history of other cancer or malignancy; infection with other hepatitis virus or HIV; a long history of alcoholism, alcoholic cirrhosis, and drug cirrhosis with a definite cause; and liver cancer caused by hepatitis C virus infection were excluded. Fasting blood samples were collected, and serum samples were obtained after centrifugation at 4000 rpm for 5 minutes. The supernatant is packed in centrifuge tubes and stored at −20°C until further use. HBV DNA was quantified through quantitative real-time PCR. The 1762/1764 mutation in the BCP region of the HBV gene was detected using the amplification refractory mutation system fluorescence PCR method, which allows us to accurately determine a single-base mutation. GGTII was detected using the double-antibody sandwich method (Fig. 1).

### 2.2. Main instruments and reagents

The MC1000 real-time fluorescence PCR analyzer was purchased from Hangzhou Dean biotechnology co., LTD. The SLAN-96P PCR instrument was purchased from group Shanghai Hongshi medical technology co., LTD., the enzyme-linked immunoanalyzer was from Shanghai branch co., LTD., the biosafety cabinet was from Shandong BIOBASE biological industry co., LTD., and the centrifuge was from Zhuhai dark horse medical co., LTD. The GGTII detection reagent was purchased from Shanghai Yansheng Biological Company. The HBV DNA quantitative testing kit was provided by Hunan Shengxiang Biotechnology Co., Ltd., and the HBV gene BCP region 1762/1764 mutation detection kit was provided by Wuhan Baitai Gene Company.

### 2.3. Statistical analysis

SPSS17.0 statistical software was used for data analysis. The Kolmogorov–Smirnov (K–S) test was conducted to determine whether the data of each group were normally distributed. The adoption rate of count data (%) was expressed, and the cross-tabular chi-square test was conducted for inter-group comparison (χ^2^). The measurement data with non-normal distribution were represented by the median (interquartile distance) (M [P25, P75]). The 3 groups were compared using the non-parametric Kruskal–Wallis test, and the 2 groups were compared using the non-parametric Mann–Whitney *U* test. The measurement data with normal distribution were expressed as mean ($\bar{x}$) ± s. One-way analysis of variance was performed for multigroup comparisons, and then, pound-for-pair multiple comparison was performed. The receiver operating characteristic (ROC) curve was used for indicator diagnosis, and ROC diagnosis, which was generated through multivariate regression analysis, was used for multi-indicator combined diagnosis. *P* < .05 was considered statistically significant.

## 3. Results

### 3.1. Comparison of general data

Through statistical analysis of biochemical indices and ratios, we found that compared with the CHB group, significant differences in serum GGT activity, GGTII levels, AST activity, AST/ALT ratio, GGT/ALT ratio, and GGT/AST ratio were observed between the 2 groups (*P* < .001, *P* = .004, *P* = 0003, *P* = .001, *P* < .001, *P* < .001, and *P* < .001). No significant difference was observed in serum ALT activity (*P* = .4). Compared with the LC group, serum GGT activity, AST activity, GGT/ALT ratio, and other indices were significantly different between the 2 groups (*P* = .001, *P* = .037, *P* = .038, respectively). No significant differences in serum ALT activity, AST/ALT ratio, GGT/AST ratio, and GGTII levels were observed between (*P* = .21, *P* = .297, *P* = .105, *P* = .186, respectively). Compared with the LC group, statistically significant differences in the AST/ALT and GGT/ALT ratios were observed between the 2 groups (*P* = .042, *P* = .021, respectively), whereas no statistically significant differences in the other indices were observed. The LogHBVDNA comparison revealed that the difference between the PLC and CHB groups was statistically significant (*P* = .023), but no statistically significant difference was observed between the PLC and LC groups and between the CHB and LC groups (*P* = .488, *P* = .129, respectively) (Table 1).

**Table 1:** General data of each study group (M [M25, M75], $\bar{x}\pm S$).

Variable	CHB group (N = 67 cases)	LC group (N = 30 cases)	PLC group (N = 48 cases)	Total *P*	*P* (CHB vs LC)	*P* (CHB vs PLC)	*P* (LC vs PLC)
ALT(U/L)	38 (21.5–111)	36 (28–70)	47.5 (29.5–72.5)	.502	.888	.4	.21
AST(U/L)	38 (27.5–87)	53 (35–92)	93 (43–141.5)	.008*	.384	.003*	.037*
GGT(U/L)	31 (21.5–78.5)	43 (31–84)	130.5 (62.5–208.5)	<.001*	.06	<.001*	.001*
AST/ALT	1.161 (0.824–1.502)	1.288 (1.157–1.795)	1.533 (1.164–2.568)	.002*	.042*	.001*	.297
GGT/ALT	0.750 (0.387–1.746)	1.221 (0.900–2.947)	2.521 (1.012–5.764)	<.001*	.021*	<.001*	.038*
GGT/AST	0.684 (0.364–1.132)	0.949 (0.630–1.550)	1.589 (0.700–2.604)	<.001*	.065	<.001*	.105
GGTII (ng/L)	206.41 ± 93.24	230.78 ± 110.15	261.67 ± 101.88	.016*	.286	.004*	.186
logHBVDNA	5 (3.435–6.335)	4.65 (3.06–5.55)	3.77 (3.08–5.49)	.052	.129	.023*	.488

ALT = alanine aminotransferase, AST = aspartate aminotransferase, CHB = chronic hepatitis B, GGT = gamma-glutamyl transpeptidase isozyme, GGTII = gamma-glutamyl transpeptidase isozyme II, LC = liver cirrhosis, PLC = primary liver cancer.

*P < .05.

### 3.2. Rate of HBV gene BCP region 1762/1764 mutation in each group

The rate of HBV gene BCP region 1762/1764 mutation between the PLC and CHB group and between the LC and CHB groups exhibited statistical significance (χ^2^ = 33.087, *P* < .001; χ^2^ = 21.061, *P* < .001, respectively), but no significant difference was observed between the PLC and LC groups (χ^2^ = 0.238, *P* = .626) (Table 2).

**Table 2:** Hepatitis B virus (HBV) gene BCP region 1762/ 1764 mutations in each study group.

Group	Number of mutation cases	Number of unmutated cases	Rate of mutation (%)
CHB	18	49	26.87%
LC	23	7	76.67%
PLC	39	9	81.25%
*P* (CHB vs LC)	*P* < .001*		
*P* (CHB vs PLC)	*P* < .001*		
*P* (LC vs PLC)	*P* = .626		

CHB = chronic hepatitis B, LC = liver cirrhosis, PLC = primary liver cancer.

*P < .05.

### 3.3. Serum GGT activity and GGTII expression levels in patients belonging to different PLC stages

The serum GGTII levels were higher in the early and late PLC groups than in the N-PLC group, but no statistical significance was observed between the early PLC and N-PLC groups (*P* = .183). By contrast, the difference between the advanced PLC and N-PLC groups was statistically significant (*P* = .006). Serum GGT activity was higher in the early and advanced PLC groups than in the N-PLC group (*P* = .001, *P* < .001). No significant differences in serum GGTII and GGT activities were observed between the early PLC and late PLC groups (*P* = .303, *P* = .390, respectively) (Table 3).

**Table 3:** Serum GGT activity and GGTII expressions of patients in different stages of the PLC groups (M [M25, M75], $\bar{x}\pm S$).

Items	N-PLC group (N = 97)	Early PLC group (N = 22)	Advanced PLC group (N = 26)	Total *P*	*P* (early PLC vs advanced PLC)	*P* (N-PLC vs early PLC) group	*P* (N-PLC vs advanced PLC)
GGTII (ng/L)	213.95 ± 98.85	245.49 ± 94.78	275.36 ± 107.45	.017*	.303	.183	.006*
GGT (U/L)	36 (24–84)	134 (42–194)	127 (75–331)	<.001*	.390	.001*	<.001*

Advanced PLC = Stage III and stage IV PLC, early PLC group = stage I and stage IIPLC, GGT: = gamma-glutamyl transpeptidase isozyme, GGTII = gamma-glutamyl transpeptidase isozyme II, N-PLC group = include CHB and LC.

*P < .05.

### 3.4. Diagnostic performance of serum GGT activity and GGTII levels for PLC and early PLC

The area under the curve (AUC) of the ROC curve of GGT and GGTII for diagnosing PLC was 0.775 (95% confidence interval [CI] [0.697, 0.854]) and 0.608 (95% CI [0.512, 0.704]), respectively. The AUC of GGT and GGTII for diagnosing early PLC was 0.732 (95% CI [0.620, 0.845]) and 0.579 (95% CI [0.452, 0.706]), respectively. GGT exhibited some good clinical diagnostic efficacy for PLC and early PLC, whereas GGTII exhibited poor clinical diagnostic performance for PLC and early PLC (Tables 4 and 5).

**Table 4:** Comparison of the diagnostic performance of the GGT and GGTII tests for PLC.

Project	AUC	Sensitivity (%)	Specificity (%)	*P* value
GGTII	0.608	89.6	32	.035*
GGT	0.732	83.3	64.9	<.001*

AUC = area under the curve, GGT = gamma-glutamyl transpeptidase isozyme, GGTII = gamma-glutamyl transpeptidase isozyme II.

*P < .05.

**Table 5:** Comparison of the diagnostic performance of GGT and GGTII tests for early PLC.

Project	AUC	Sensitivity (%)	Specificity (%)	*P* value
GGTII	0.579	81.8	32.0	.570
GGT	0.732	63.6	82.5	.001*

AUC = area under the curve, GGT = gamma-glutamyl transpeptidase isozyme, GGTII = gamma-glutamyl transpeptidase isozyme II.

*P < .05.

### 3.5. Diagnostic performance of the BCP region 1762/1764 mutation combined with serum GGT and GGTII activities for PLC and early PLC

Considering the N-PLC group (CHB group, LC group) as the control group and the PLC group as the case group, BCP 1762/1764 mutation was assigned “1” and no mutation was assigned “0.” The ROC curve of the BCP region 1762/1764 mutation combined with GGT and GGTII for PLC diagnosis, and the AUC of the BCP region 1762/1764 mutation combined with GGT and that combined with GGT and GGTII were 0.816 (95% CI [0.743, 0.888]) and 0.818 (95% CI [0.743, 0.892]), respectively. The ROC curve of the BCP region 1762/1764 mutation combined with GGT and GGTII for early PLC diagnosis, and the AUC of the BCP region 1762/1764 mutation combined with GGT and that combined with GGT and GGTII was 0.816 (95% CI [0.726, 0.906]) and 0.815 (95% CI [0.725, 0.902]), respectively. The combination of GGT and the HBV gene BCP region 1762/1764 mutation was of some clinical diagnostic value for both PLC and early PLC. However, the combined index GGTII was no diagnostic value for PLC and early PLC (Tables 6 and 7).

**Table 6:** Comparison of the diagnostic performance of the hepatitis B virus (HBV) gene in the BCP region 1762/1764 combined with the GGT and GGT test for PLC.

Project	AUC	Sensitivity (%)	Specificity (%)	*P* value
BCP region 1762/ 1764 + GGT	0.816	77.1	75.3	<.001*
BCP region 1762/ 1764 + GGTII + GGT	0.818	66.7	89.7	<.001*

AUC = area under curve, BCP = basal core promoter, GGT = gamma-glutamyl transpeptidase isozyme, GGTII = gamma-glutamyl transpeptidase isozyme II.

*P < .05.

**Table 7:** Comparison of the diagnostic performance of the hepatitis B virus (HBV) gene in the BCP region 1762/1764 combined with GGT and GGT tests for early PLC.

Project	AUC	Sensitivity (%)	Specificity (%)	*P* value
BCP zone 1762/ 1764 + GGT	0.816	72.7	77.3	<.001*
BCP zone 1762/ 1764 + GGTII + GGT	0.815	72.7	79.4	<.001*

AUC = area under curve, BCP = basal core promoter, GGT ^=^ gamma-glutamyl transpeptidase isozyme, GGTII = gamma-glutamyl transpeptidase isozyme II.

*P < .05.

## 4. Discussion

HBV continues to infect patients, thereby severely affecting their life and health. HBV pathogenicity is related to the patient disease itself, immunity function, and HBV virulence. At present, many drugs are available for HBV infection treatment, but completely eliminating the virus is difficult.^[10]^ When the treatment is discontinued, the HBV may continue to replicate and rebound. PLC is currently difficult to cure clinically or has a poor prognosis after treatment, and thus causes great harm to patients. The chronic infection caused by HBV is closely related to the occurrence of liver cancer.

GGT is present in the tissue cells of various organs, but serum GGT is mainly derived from the hepatobiliary system. When hepatocytes are damaged or the liver is blocked, serum GGT levels increase.^[11]^ GGTII is an isoenzyme of GGT, and GGT is less specific in PLC diagnosis, while some studies have shown the high specificity of GGTII, with a high positive rate observed in liver cancer, whereas a low positivity observed in the normal population or other diseases.^[12]^ This study revealed that differences in serum GGT activity, AST activity, GGT/ALT ratio, and GGT/AST ratio were observed between the PLC and CHB and the LC groups, which is similar to the results of relevant studies conducted at home and abroad. The serum GGTII level increased in the PLC group. The difference in serum GGTII levels between the PLC and CHB groups was statistically significant, but the difference between the LC and LC groups was statistically nonsignificant. Meanwhile, serum GGTII levels in the early and advanced PLC groups were higher than those in the N-PLC group, but no statistical significance was observed between the early PLC and N-PLC groups, whereas specific statistical significance was observed between the advanced PLC and N-PLC groups. According to the results, GGTII was unsuitable as a marker for early PLC, but it may be an effective indicator for monitoring the treatment and recurrence of advanced hepatitis B PLC. At the same time, we observed that serum GGT activity in the early PLC and advanced PLC groups was higher than that in the N-PLC group. These results indicate that the levels of the GGT isoenzyme GGTII also increased in the early stage of liver cancer, but the increase was not significant compared with that in the N-PLC group (LC group and CHB group), which is inconsistent with the results of some domestic and foreign studies. The possible reasons are that all patients with hepatitis B are inconsistent with those of other studies. Furthermore, although GGT levels are increased in patients with early liver cancer, more specific markers other than GGTII may exist.

In this study, the HBV gene BCP region 1762/1764 mutation was detected using the amplification refractory mutation system fluorescence PCR method. This method can accurately detect the presence of the mutant strain HBV virus and can help understand the situation of HBV mutation at an early stage. This detection method is fast and convenient.^[13]^ Other relevant studies have shown that the presence of HBV gene BCP region 1762/1764 mutation in chronic HBV-infected patients is a high-risk factor for PLC development.^[14]^ These mutations can inhibit the expression of viral HBeAg, but HBV replication may not be hindered. Low viremia in CHB patients is closely related to liver fibrosis and liver cancer.^[15,16]^ Here, we showed that HBV DNA levels were lower in the PLC group than in the CHB group, indicating that low viremia continuously damages the liver and its immune function. The rate of BCP 1762/1764 mutation was higher in the PLC group than in the CHB group, but no difference was observed between the PLC and LC groups, which indicated that the HBV gene BCP region 1762/1764 mutation in CHB-infected patients may be closely related to PLC or LC.

HBV gene mutation combined with GGT has been to have a certain diagnostic value for liver cancer.^[17]^ In the present study, the ROC curve of the HBV gene mutation and GGT and GGTII for PLC and early PLC diagnosis revealed that GGT had a good clinical diagnostic efficiency for PLC and early PLC, whereas GGT exhibited poor clinical diagnostic performance for PLC and early PLC. The HBV gene BCP region 1762/1764 mutation combined with GGT exhibited better diagnostic performance for PLC and early PLC than the single index. The study indicate that the combined analysis of HBV gene mutation and GGT can improve their diagnostic performance for PLC and early PLC, which may become a crucial indicator for the early diagnosis of PLC. The combined index GGTII did not exhibit improved diagnostic performance for PLC and early PLC. Therefore, the index GGTII is not a good choice for early PLC monitoring.

The PLC pathogenesis is complex and many factors are involved, so early PLC diagnosis generally requires a combination of multiple indicators. In the present study, patients with CHB infection were screened for the HBV gene mutation for the stratified management of hepatitis B patients. This screening was then combined with the determination of biochemical indicators, namely GGT and GGTII. This combination if used as a regular screening technique would allow possible earlier detection, diagnosis, and treatment of liver cancer, thereby benefitting the health of CHB patients.

The main study limitations are as follows: the study participants are patients infected with chronic HBV, including those with CHB, LC, or PLC. These patients reflect a disease state in the HBV infection process, and relevant studies on statistics and diagnostic tests can be conducted. However, when HBV mutations occur in the BCP region, whether the disease deteriorates after mutations, or whether the mutation occurs as the disease progresses remains unclear. Therefore, these questions need to be addressed, and the next step is to conduct prospective studies on patients with hepatitis B infection to answer these questions.

## Author contributions

**Conceptualization:** Shunhua Qiu, Dewen Zhang.

**Data curation:** Shunhua Qiu, Dan Yang.

**Formal analysis:** Shunhua Qiu.

**Funding acquisition:** Shunhua Qiu, Dewen Zhang.

**Investigation:** Shunhua Qiu, Dan Yang, Dewen Zhang.

**Methodology:** Shunhua Qiu, Dewen Zhang.

**Project administration:** Shunhua Qiu, Dewen Zhang.

**Resources:** Shunhua Qiu, Dewen Zhang.

**Software:** Shunhua Qiu, Lifen Jin.

**Supervision:** Shunhua Qiu, Dewen Zhang.

**Validation:** Shunhua Qiu, Dewen Zhang.

**Visualization:** Shunhua Qiu, Dewen Zhang.

**Writing – original draft:** Shunhua Qiu.

**Writing – review & editing:** Shunhua Qiu.
